# Transient Aortic Occlusion Augments Collateral Blood Flow and Reduces Mortality During Severe Ischemia due to Proximal Middle Cerebral Artery Occlusion

**DOI:** 10.1007/s12975-015-0443-5

**Published:** 2015-12-26

**Authors:** Gomathi Ramakrishnan, Bin Dong, Kathryn G. Todd, Ashfaq Shuaib, Ian R. Winship

**Affiliations:** Neurochemical Research Unit, Department of Psychiatry, Faculty of Medicine and Dentistry, University of Alberta, Edmonton, AB Canada; Neuroscience and Mental Health Institute, University of Alberta, 12-127 Clinical Sciences Building, Edmonton, AB Canada T6G 2R3; Division of Neurology, Department of Medicine, Faculty of Medicine and Dentistry, University of Alberta, Edmonton, AB Canada

**Keywords:** Stroke, Collateral blood flow, Ischemia, Neuroprotection, Collateral therapeutics, Cerebral blood flow

## Abstract

Cerebral collateral circulation provides alternative vascular routes for blood to reach ischemic tissues during stroke. Collateral therapeutics attempt to augment flow through these collateral channels to reduce ischemia and brain damage during acute ischemic stroke. Transient aortic occlusion (TAO) has pre-clinical data suggesting that it can augment collateral blood flow and clinical data suggesting a benefit for patients with moderate cortical strokes. By diverting blood from the periphery towards the cerebral circulation, TAO has the potential to augment primary collateral flow at the circle of Willis and thereby improve outcome even during large, hemispheric strokes. Using proximal middle and anterior cerebral artery occlusion in rats, we demonstrate that TAO reduces mortality and improves collateral blood flow in severely ischemic animals. As such, TAO may be an effective therapy to reduce early mortality during severe ischemia associated with proximal occlusions.

Collateral circulation refers to pre-existing vascular redundancies that provide a route for blood to reach a target tissue when a primary channel is blocked [1–5]. Primary cerebral arterial collaterals refer to short arterial segments in the circle of Willis that allow blood flow between the territories of the internal carotid arteries and the vertebrobasilar system or between cerebral hemispheres in the event of proximal occlusion. The secondary cerebral collaterals include the pial (or leptomeningeal) collaterals, which are anastomotic connections located on the surface of the cortex that connect distal branches of the anterior, middle, and posterior cerebral arteries (ACA, MCA, PCA). These collateral channels permit blood flow from the territory of an unobstructed artery into the territory of an occluded artery (e.g., retrograde filling of the MCA territory via anastomoses with the ACA after middle cerebral artery occlusion (MCAo)) [2–4, 6]. Collateral extent is crucial, as in both animal models and human stroke patients the degree of collateral perfusion during cerebral ischemia is a predictor of stroke severity, prognosis, and response to reperfusion therapy [7–15].

“Collateral therapeutics” augment blood flow through collaterals to improve perfusion of penumbral tissue during acute ischemic stroke. Recently, promising pre-clinical and clinical data support the use of transient aortic occlusion (TAO) to increase global cerebral perfusion and reduce damage due to stroke. Pre-clinical work in TAO-treated rats has demonstrated reduced infarct size [16] and augmentation of flow through pial collaterals due to TAO after thromboembolic MCAo [17]. Data from the Safety and Efficacy of NeuroFlo Technology in Ischemic Stroke (SENTIS) trial suggests that TAO is safe and improves stroke outcome in subsets of stroke patients [4, 16, 18–21]. Patients with cortical ischemic stroke presenting within 5 h of onset, with National Institutes of Health Stroke Scale (NIHSS) between 8 and 14 (moderate severity), and patients older than 70 years of age showed the greatest benefit from TAO [19]. Additional analysis of the SENTIS trial reports a significant reduction in stroke-related mortality and severe disability with TAO [22]. Notably, the mortality difference was concentrated in the patients at highest risk (NIHSS scores >14 and those older than age 70) [19, 20]. This reduction in stroke-related mortality in patients with severe stroke suggests that TAO may be beneficial for patients with proximal occlusions resistant to intravenous thrombolysis, such as internal carotid occlusions or proximal occlusion of the MCA [23, 24]. Here, we evaluated the efficacy of TAO to improve collateral blood flow and reduce mortality in a model of proximal MCA/ACA occlusion.

## Methods

Experimental protocols conform to the guidelines established by the Canadian Council on Animal Care and were approved by the Health Sciences Animal Care and Use Committee at University of Alberta. Urethane-anaesthetized (i.p., 1.5 g/kg in sterile saline) male Sprague Dawley rats (400–450 g, 3–4 months of age) divided into two treatment groups (MCAo + TAO, *n* = 10; MCAo + Sham TAO, *n* = 11) underwent laser speckle contrast imaging (LSCI) through a thinned-skull imaging window. LSCI maps of blood flow were acquired at baseline, post-MCAo, 15 min after cessation of TAO (or sham, 2 h post-MCAo), and 75 min after TAO (or sham, 3 h post-MCAo). Immediately after the final imaging session, animals were euthanized and their brains were removed. Early indices of infarct size and location were assessed on cryostat-sectioned 20-μm coronal brain sections stained with hematoxylin and eosin (H&E). Animals that did not survive through all imaging sessions (4, all from the sham-TAO group) or with poor image quality (1, due to degradation of the optical window) were excluded from LSCI analyses.

### LSCI

LSCI was performed via a ~5 × 5-mm thin skull cranial window over the distal regions of the MCA territory [6, 17, 25]. Back-scattered light was collected by a Dalsa 1M60 Pantera CCD camera (5 ms exposure time) during illumination with a 784-nm (32 mW) laser (StockerYale, Inc.). Speckle contrast (*K* = σ_s_/*I*) was determined using ImageJ software (NIH) [6, 26]. Maps of *K* show the pattern of blood flow on the cortical surface during imaging, with darker vessels demonstrating relatively faster blood flow than lighter vessels. *K* values were converted to correlation times (τ_c_) that are approximately inversely proportional to blood flow velocity [17, 26, 27]. Data are expressed as *τ*_*Baseline*_*/τ*_*c*_ or *τ*_*MCAo*_*/τ*_*c*_ values that illustrate changes in blood flow from baseline or post-MCAo values, respectively. Vessel diameters were determined using an ImageJ plug-in that uses a full width at half-maximum algorithm [17, 28].

### Filament Occlusion

To induce proximal occlusions of the MCA and ACA, the right common, internal, and external carotid arteries (CCA, ICA, ECA) were exposed. A silicon rubber-coated monofilament (Doccol Corporation, filament size 3-0, diameter 0.20 mm, length 30 mm; diameter with coating 0.54+/− 0.02 mm; coating length 5–6 mm) was advanced through the ECA into the ICA until lodging proximal to the origin of MCA and ACA and then sutured in place.

### Transient Aortic Occlusion

To transiently occlude the descending aorta [16, 17], a dilation catheter (2.0 mm diameter, Cordis Fire Star RX PTCA) was advanced past the origin of the renal artery from the femoral artery. Aortic flow diversion was initiated 60 min after ischemic onset and maintained for 45 min.

## Results

**Fig. 1:**
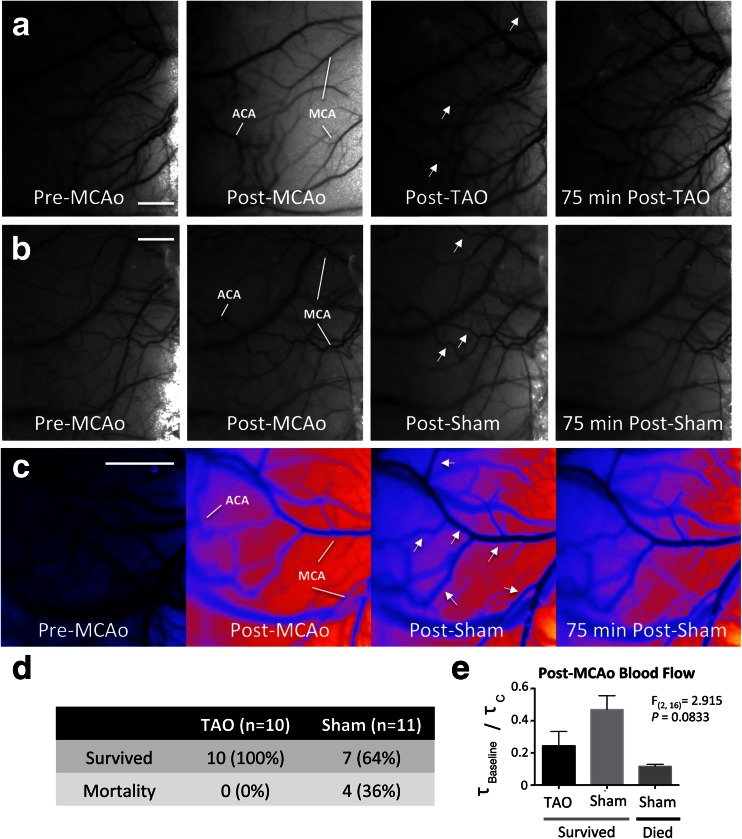
**a** Laser speckle contrast imaging (LSCI) data showing changes in blood flow associated with proximal middle cerebral artery occlusion (MCAo) and treatment with transient aortic occlusion (TAO). Images show blood flow recorded at baseline (pre-MCAo), post-MCAo, 15 min after TAO, and 75 min after TAO. Anastomoses between the MCA and anterior cerebral artery (ACA) are apparent after MCAo. An increase in speckle contrast intensity post-MCAo reflects a decrease in blood flow (ischemia), while the *darker images* after TAO demonstrate increased blood flow after treatment. **b** Representative LSCI data from a sham-TAO animal showing a more moderate ischemia (less intense speckle contrast, but clear formation of anastomoses indicating MCAo) and little change in blood flow after sham. **c** Pseudo-colored LSCI maps of blood flow (with *cooler colors* reflecting greater blood flow, *warmer colors* showing lower flow) showing increases in flow through MCA segments downstream of ACA connections (see *white arrows*) after TAO (note increased diameter and *darker colors* of identified MCA segments). **d** Contigency table illustrating reduced mortality prior to imaging completion (within 3 h of MCAo onset) in animals treated with TAO as opposed to sham-TAO. **e** *τ*_*Baseline*_ */τ*_*c*_ values reflecting changes in blood flow velocity relative to baseline show that TAO animals that survived through imaging had a trend towards lower blood flow than did sham-TAO animals that survived. Sham-TAO animals that died within 3 h of MCAo onset had more severe ischemia (reduced *τ*_*Baseline*_ */τ*_*c*_), suggesting that lower mean *τ*_*Baseline*_ */τ*_*c*_ in TAO animals results from reduced mortality in severely ischemic animals. *Scale bars*, 1 mm

LSCI was performed to monitor the blood flow in cortical surface vessels during proximal MCA and ACA occlusion followed by TAO (*n* = 10) or Sham-TAO (*n* = 11). Consistent with previous studies, anastomoses between distal ACA and MCA segments were apparent after MCAo but not during baseline imaging (Fig. 1a, b, white arrows). Qualitative analysis of LSCI data showed clear decreases in blood flow by MCAo and increased flow that persists after TAO in some but not all TAO-treated rats (Fig. 1a, c; note an increase in speckle contrast after MCAo that reflects a reduction in flow and a darkening of the image as blood flow increases due to TAO). Arrows in Fig. 1c show regions of drastic increases in flow in MCA segments downstream of ACA-MCA anastomoses in a TAO-treated animal. In other animals (from both treatment groups), reductions in flow after MCAo were less drastic and distinct changes due to TAO or sham were not apparent (Fig. 1b). Notably, early mortality (between 1 and 3 h after ischemic onset, prior to completion of the final imaging session 3 h after ischemic onset) was significantly reduced in TAO-treated rats relative to Sham-TAO (Fig. 1d, *χ*^2^(2) = 4.49, *P* = 0.034). Analysis of blood flow (*τ*_*Baseline*_*/τ*_*c*_) in distal MCA segments downstream of ACA anastomoses after MCAo but prior to treatment (or sham) suggests that early mortality occurred in Sham-TAO rats with severe ischemia (Fig. 1e). Multivariate analyses of physiological parameters monitored via pulse oximetry (Table 1) did not reveal any differences in oxygen saturation, heart rate, or breath rate that could account for differences in early mortality.

**Table 1:** Physiological parameters

		Oxygen saturation	Heart rate	Breath rate
Pre-MCAo				
TAO	Mean	98.83	366.75	100.43
	S.E.M	0.38	10.69	5.92
Sham—survived	Mean	99.09	386.76	97.55
	S.E.M	0.34	20.66	6.80
Sham—died	Mean	99.45	365.26	85.22
	S.E.M	0.05	22.84	7.61
Post-MCAo				
TAO	Mean	98.84	389.54	96.53
	S.E.M	0.14	11.91	6.56
Sham—survived	Mean	99.18	414.15	89.58
	S.E.M	0.22	20.28	3.22
Sham—died	Mean	99.56	394.81	86.03
	S.E.M	0.05	22.84	7.61
Post-TAO or sham				
TAO	Mean	95.98	404.40	96.99
	S.E.M	0.93	13.15	5.68
Sham—survived	Mean	98.20	381.01	94.99
	S.E.M	1.08	31.18	8.85

Multivariate analyses of physiological parameters monitored via pulse oximetry did not reveal any differences in oxygen saturation, heart rate, or breath rate that could account for differences in early mortality

**Fig. 2:**
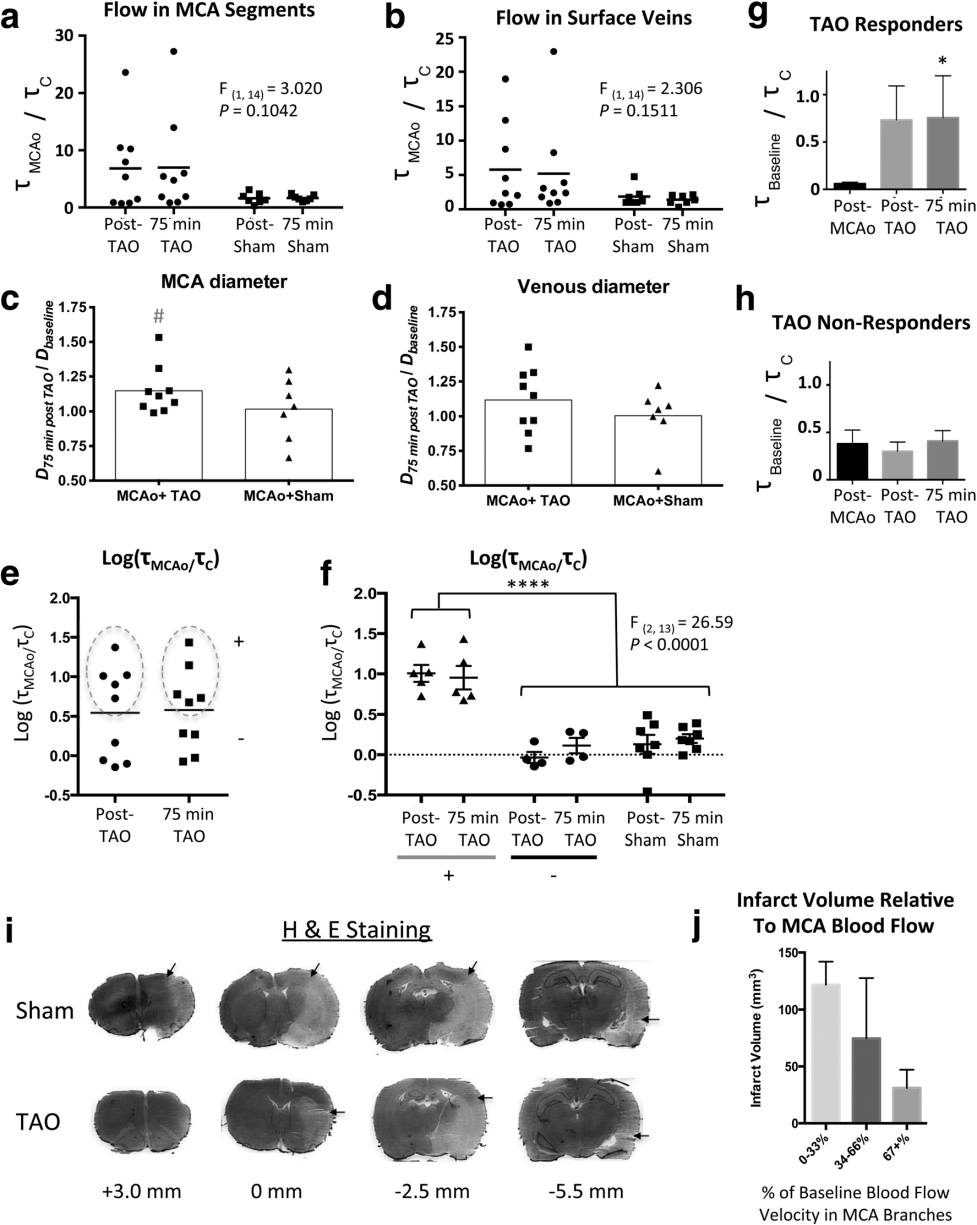
**a**, **b** Scatter plots showing *τ*_*MCAo*_ */τ*_*c*_ values measured in distal MCA segments or surface veins, respectively, for individual rats after middle cerebral artery occlusion (MCAo) and transient aortic occlusion (TAO) or sham-TAO. The *horizontal line* denotes the mean. While multivariate ANOVA did not identify a significant main effect, significant increases in blood flow from post-MCAo values were observed in some (5/9) TAO-treated rats. No comparable changes in flow were observed in sham-TAO rats. **c**, **d** Diameter of distal MCA segments and surface veins, respectively, measured 75 min after cessation of TAO or Sham-TAO. # denotes a significant difference from a hypothetical (normalized) baseline value of 1.0 on a single sample *t* test. **e**, **f** A variance-stabilizing transformation was achieved by performing a log transform of *τ*_*MCAo*_ */τ*_*c*_ values. **e** shows a clear separation among rats that responded to TAO with an increase in *τ*_*MCAo*_ */τ*_*c*_ in distal MCA segments (+, denoted by *dashed oval*) and rats that did not respond to TAO. **f** Multivariate ANOVA detected a significant effect of group on log(*τ*_*MCAo*_ */τ*_*c*_) when rats were categorized as TAO “responding” or “non-responding” (i.e., rats that exhibited a clear increase in MCA flow after TAO vs. rats with no significant change in MCA flow) or sham-TAO. **** denotes significant (*P* < .0001) Holm-Sidak post hoc comparisons. **g**, **h** *τ*_*Baseline*_ */τ*_*c*_ values from TAO responders and non-responders, respectively, showing that TAO responders had severe ischemia prior to treatment that was normalized near baseline values by TAO (whereas TAO non-responders had less severe ischemia and no change due to treatment). **i** Representative H&E-stained tissue sections showing demarcation of early infarct from healthy tissue (*black arrows*, distances from Bregma shown below images). **j** Pooling *τ*_*Baseline*_ */τ*_*c*_ values across treatment groups, a clear inverse relationship between amount of blood flow (relative to baseline) in distal MCA segments (measured 75 min after TAO or sham-TAO, 2 h after MCAo onset) and the volume of early infarct detected 3 h after MCAo onset

Because early mortality resulted in group differences in post-MCAo blood flow, *τ*_*c*_ were normalized to post-MCAo values within each animal to examine treatment-induced changes in blood flow in distal MCA segments and in surface veins draining the ischemic territories (Fig. 2a, b). Multivariate ANOVA did not reveal a significant main effect of treatment on *τ*_*MCAo*_*/τ*_*c*_ (MCA segments, *F*_(1, 14)_ = 3.020, *P* = 0.1042; veins, *F*_(1, 14)_ = 2.306, *P* = 0.1511). However, a clear trend towards augmented collateral flow in MCA segments was apparent in a subset of TAO-treated rats. Notably, drastic increases in flow after TAO did not occur in any Sham-TAO animals. TAO “responders” were therefore defined as animals in which *τ*_*MCAo*_*/τ*_*c*_ was greater than the mean *τ*_*MCAo*_/*τ*_*c*_ from sham animals plus two standard deviations [5/9 (56 %) of rats in the TAO group met this criteria; see Fig. 1c for representative speckle contrast images showing increased flow]. Vessel diameter (normalized to baseline diameter, measured 75 min after cessation of TAO or Sham-TAO) was not significantly different between treatment groups in MCA segments or surface veins (Fig. 2c, d) (unpaired *t* test, *P* > .05). However, single sample *t* tests (hypothetical baseline of 1.0) suggest that MCA segments were significantly dilated relative to baseline only in the TAO group (*P* = .032).

### TAO Responders Versus Non-responders

A variance-stabilizing log transformation of MCA flow ([log(*τ*_*MCAo*_*/τ*_*c*_)], Fig. 2a) was performed to better visualize response patterns. Figure 2e shows log(*τ*_*MCAo*_*/τ*_*c*_) data from MCA segments in TAO-treated rats, indicating a clear separation of rats who responded to TAO with an increase in collateral flow (+, denoted by dashed line) or did not respond to treatment (−). Multivariate ANOVA of log(*τ*_*MCAo*_*/τ*_*c*_) for TAO “responders,” TAO “non-responders,” and Sham-TAO rats (Fig. 2f) revealed a significant main effect of group (*F*_(2, 13)_ = 26.59, *P* < 0.0001), with Holm-Sidak post hoc comparisons demonstrating that TAO responders had significantly greater increases in log(*τ*_*MCAo*_*/τ*_*c*_) relative to TAO non-responders or sham animals (*P* < .0001). Notably, TAO responders had very low blood flow after MCAo (*τ*_*Baseline*_*/τ*_*c*_ = 0.06 ± 0.016 or ~6 % of baseline) and showed a drastic increase after TAO (Fig. 2g). Non-responders had more moderate ischemia (*τ*_*Baseline*_*/τ*_*c*_ = 0.38 ± 0.14 or ~38 % of baseline) and no change after TAO (Fig. 2h). Friedman’s repeated measures tests on *τ*_*Baseline*_*/τ*_*c*_ in responders and non-responding animals confirmed a statistically significant relationship between TAO and blood flow in TAO responders (*χ*^2^(3) = 7.60, *P* = .0239) but not non-responders (*P* > .05).

### Histology

Early infarct was visualized using H&E staining (Fig. 2i). Infarct volume was 93.84 ± 21.92 mm^3^ in TAO-treated rats and 107.1 ± 36.89 mm^3^ in Sham-TAO rats (unpaired *t* test, *P* > .05). While difference in pre-treatment but post-MCAo flow in animals that survived to 3-h post-MCAo makes comparisons between treatment groups difficult, plotting infarct volume as a function of blood flow (*τ*_*Baseline*_*/τ*_*c*_) in MCA segments 75 min after TAO or Sham-TAO illustrates a clear inverse relationship between collateral flow and early infarct volume (Fig. 2j).

## Discussion

In the SENTIS trial, 515 patients with cortical ischemic stroke received 45 min of TAO or standard stroke treatment. SENTIS data suggest that TAO is safe and may be an effective therapy for subsets of stroke patients [18–20, 22, 29]. These trials in cortical stroke patients suggested maximal treatment efficacy for those with moderate stroke severity [19]. By redistributing blood towards the head from the peripheral circulation, TAO has the potential to augment both primary (circle of Willis) and secondary (pial) collateral flows [1, 2]. Here, using a large filament-based occlusion to block the origins of the MCA and ACA, we evaluated whether TAO could improve collateral blood flow and reduce mortality during severe stroke. Because this model would occlude the origins of the ACA and MCA, enhanced collateral flow through the circle of Willis (i.e., through the anterior and/or posterior communicating arteries) would be required to reduce ischemia and mortality. Our data show a significant reduction in early mortality with TAO. Moreover, LSCI data suggest that in animals with severe ischemia, TAO can improve flow in MCA segments downstream of ACA anastomoses and reduce ischemia. In animals with more moderate ischemia, TAO did not induce significant increases in blood flow. This may suggest that in animals or patients with effective primary collaterals, TAO does not further enhance flow. However, in severe ischemia associated with poor primary collateral flow and high mortality [30], TAO may be an effective acute therapy to maintain blood flow to ischemic regions at a level sufficient to prevent early mortality.

While promising, it is important to note that our measure of early mortality may not reflect total mortality outside the acute period. Previous reports suggest that mortality after permanent MCAo in Sprague Dawley rats results in the days following ischemic onset rather than the first few hours [31]. As such, our early mortality was surprising. However, our occlusion model used a large calibre (~0.54 mm including coating) filament to block carotid flow to the ACA and MCA, and this severe ischemia may account for the early mortality. Consistent with this interpretation, mortality of 13 % in the first 24 h has been reported after MCAo with 0.193-mm diameter filaments, whereas smaller filaments (0.180 mm) produced smaller infarcts and did not result in mortality in this period [32]. We observed increased TAO efficacy (greater increase in flow relative to post-stroke levels) in rats with severe ischemia prior to treatment, and early mortality may result from this severe ischemia in untreated rats. However, while we postulate a mechanism involving augmentation of primary collateral flow (via the circle of Willis), this was not verified by our imaging (which measured flow in surface arteries downstream of any increases in primary collateral flow). Nonetheless, the current data suggests that TAO warrants further investigation as stand-alone neuroprotective strategy to reduce acute mortality after severe stroke and as an adjunct therapy to maintain tissue viability during treatment of severe ischemic stroke.
